# Supplementary material for: Alterations in sperm long RNA contribute to the epigenetic inheritance of the effects of postnatal trauma
Source: Mol Psychiatry. 2018 Oct 30;25(9):2162–74. doi: 10.1038/s41380-018-0271-6 (PMC7473836; doi:10.1038/s41380-018-0271-6)
Supplement: Supplementary file 1 — Supplementary Material [file 41380_2018_271_MOESM1_ESM.docx]

**Supplementary Material**

**Alterations of sperm long RNA contribute to the epigenetic inheritance of the effects of postnatal trauma**

K. Gapp^1,2,3^, G. van Steenwyk^4^, P.L. Germain^4^, W. Matsushima^1,2,3^, K.L.M. Rudolph^1,2,3^, F. Manuella^4^, M. Roszkowski^4^, G. Vernaz^1,2,3^, T. Ghosh^1,2,3^, P. Pelczar.^5^, I.M. Mansuy^4,^*, E.A. Miska^1,2,3,^*

**Supplementary figure 1 (Gapp et al)**

**.**  ****

**Effect of MSUS on behavior.** F1 males exposed to MSUS (a) have significantly reduced latency to first enter an open arm on an elevated plus maze (Mann–Whitney U=437.5, F1 controls n=31, F1 MSUS n=46, p<0.01) and (b) covered a longer distance on the maze (Mann–Whitney U = 446, F1 controls n=31, F1 MSUS n=46, p<0.01. (c) They spent significantly more time in the bright compartment of a light dark box (F1 controls n=34, F1 MSUS n=43, t(74,14)=-2.01, p<0.05) independent of (d) their latency to first enter the dark compartment (Mann–Whitney U=681, F1 controls n=34, F1 MSUS n=43, p>0.05). They also (e) showed a tendency to spent significantly more time floating on the forced swim test (F1 controls n=36, F1 MSUS n=47, t(1.69)=81 p=0.09). Data are median ± whiskers. *p<0.05, **p<0.01, t-test or Mann-Whitney test, as appropriate. Boxes and triangles: values that lie outside the sum of the 75^th^ percentile and 1.5 x the interquartile range or the 25^th^ percentile minus 1.5 x the interquartile range (all values included in statistical analysis)..

**Supplementary figure 2 (Gapp et al)**

**Size distribution of sperm RNA used for injections into fertilized oocytes**. (a) Bioanalyzer profiles of size-selected small (<200 nt) and long (>200 nt) RNA purified from sperm samples, each being a pool from 4 males/group, showing well-segregated size populations for both control (co) and MSUS (MS) sperm RNA. (b) Bioanalyzer profiles of size-selected long RNA after fragmentation showing a clear shift of size populations to values below 200 nt for both control and MSUS sperm RNA.

**Supplementary figure 3 (Gapp et al)**

**Effects of the injection of sperm long and small RNA into zygotes on behavior of the resulting animals when adult**. Injection of small RNA (<200 nt) of F1 MSUS males into naïve zygotes affects (a) the total distance moved on an elevated plus maze (Small RNA of controls n=25, small RNA of MSUS n=20, t(43)=2.96, **p<0.01; Long RNA of controls n=17, Long RNA of MSUS n=22, t(37)=0.48, p>0.05) but (b) not the latency to first enter the dark compartment of a light dark box (Small RNA of controls n=24, small RNA of MSUS n=17, Mann Whitney U=182; p>0.05; Long RNA of controls n=18, Long RNA of MSUS n=21, Mann Whitney U=171.5 p>0.05) in the resulting male offspring when adult. Data are median ± whiskers. Dots, boxes, triangles and circles: values that lie outside the sum of the 75^th^ percentile and 1.5x the interquartile range (all values included in statistical analysis)..

**Supplementary figure 4 (Gapp et al)**

**RNA composition revealed by next generation sequencing in sperm, zygotes and 4-cell embryos.** Transcriptome of sperm and zygotes of control and MSUS males, zygotes resulting from mating of control or MSUS males with naïve control females, and 4-cell embryos resulting from the injection of fragmented long RNA from sperm of control or MSUS males into naïve control zygotes reveals variable levels of transcripts mapping to mitochondrial ribosomal RNA (Mt rRNA), ribosomal RNA (rRNA), protein coding RNA (protein-coding), long intergenic non-coding RNA (lincRNA), repeat elements (repeat elements) and others. Sperm libraries first batch: a; second batch: b are technical replicates.

**Supplementary figure 5 (Gapp et al)**

**Integrity of sperm long RNA transcripts**. Overall mapping of sequencing reads across normalized length of long sperm transcripts shows consistent coverage across all exons.

**Supplementary figure 6 (Gapp et al)**

**Principal component (PC) analysis of next-generation sequencing experiment.** Reads mapping to any class of long RNA shows clustering of RNA populations according to treatment (Control or MSUS) (principal component 1). Sperm libraries first batch: a; second batch: b are technical replicates.

**Supplementary figure 7 (Gapp et al)**

**Assessment of the impact on MSUS sperm differential expression of different ways to analyze the technical replicates.** While the different libraries created from each sample do not represent full biological replicates, it helps to estimate part of the technical variability, and we therefore compared the robustness of the main results when trying different approaches to account for this. Each dot represents a gene tested for differential expression, colored according to the density of points. The x-axis indicates the FDR when using all samples in the standard fashion (as done for Figure 4 and described in the methods), while the y-axes indicate the corresponding FDRs when using only the second set of libraries (panel a), or when using a random effect variable to account for the nested replication model (panel b; the exact model used was ~batch+(1|sample)+group). Both approaches correlate well with the standard analysis used in Figure 4.

**Supplementary figure 8 (Gapp et al)**

**a**

**b**

**Differential expression of transposable elements (TEs) in sperm of control and MSUS animals.** (a) When normalized to all transcripts, the vast majority of TEs appear downregulated in MSUS (only TEs are plotted). (b) When TEs are normalized separately from other RNA types (assuming that the global difference in TEs is technical, and that most TEs are in similar amount in the two groups), some TEs instead are upregulated.

**Supplementary figure 9 (Gapp et al)**

**.**
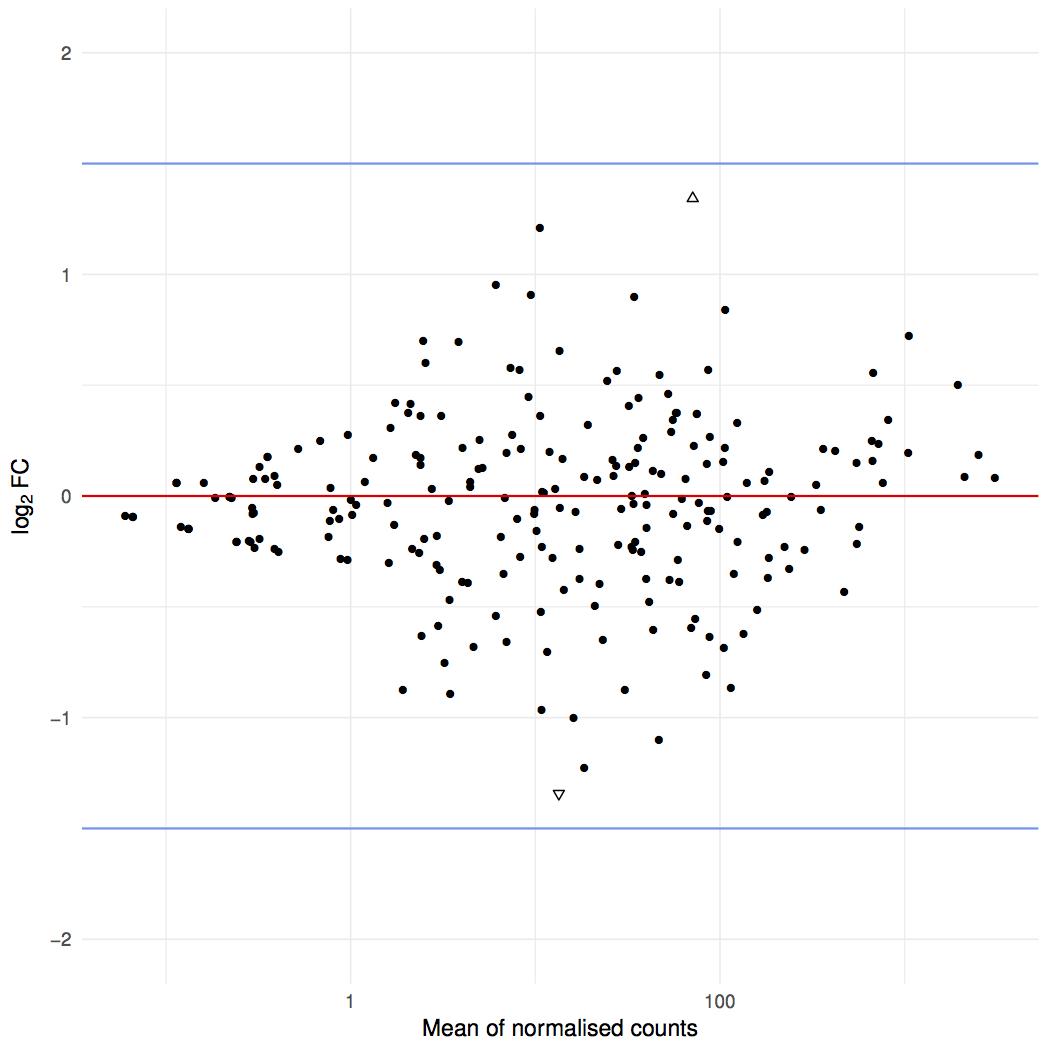


**Effect of MSUS on sperm tRNA fragments.** Analysis of next generation sequencing data reveals no change in the expression of tRNA fragments in animals exposed to MSUS (Control n=3, MSUS n=3, each sample represents pooled RNA from the sperm of 5 males, 3 biological replicates/group). Data used for this analysis have been published previously^1^.

**Supplementary figure 10 (Gapp et al)**

**Effect of MSUS on gene expression in the offspring at zygotic stage**. GO term analyses of differentially expressed genes in F2 control and MSUS zygotes shows that 18 genes are affected by MSUS (multiple testing corrected, p<0.05). Each library reflects a pool of 7-10 zygotes (Control n=4 and MSUS n=4). Top most specific GO terms enriched among differentially expressed genes are related to reproduction, but also include import into cell and neurotransmitter uptake. Red bars represent p-values of each GO term enrichment after multiple comparison correction. Blue bars represent fold enrichment of each GO term. Each blue square depicts a differentially expressed sperm transcript. Shades of blue represents the significance of its differential expression. ob= obsverved, exp= expected.

**Supplementary figure 11 (Gapp et al)**

a b

**Gene expression in zygotes and 4-cell embryos revealed by next generation sequencing.** (a) Genes with a trend (p<0.01 before correction for multiple testing) in 4-cell embryos injected with fragmented MSUS sperm RNA vs fragmented control sperm RNA do not show any similar trend in zygotes from natural MSUS and controls. (b) Expression of protein coding and long non-coding transcripts identified as differentially expressed in MSUS or control zygotes show no difference between 4-cell embryos resulting from zygotes injected with fragmented long RNA from MSUS vs control sperm (n=4 for each group, each library reflecting a pool of 7-10 embryos).

Supplementary table 1

(separate file) GO term enrichment analysis of control sperm RNAs.

**Supplementary table 2**

(separate file) GO term enrichment of differentially expressed genes between controls and MSUS (sheet a) including enrichments specific to up-regulated and down-regulated genes (sheets b and c), as well as similar enrichments for analyses including only the second library batch (sheet d) or using a random effect variable to account for the relatedness of technical replicates (sheet e).

**Supplementary table 3**

(separate file) GO term enrichment analysis of differentially expressed genes in MSUS zygotes.

**Supplementary table 4**

(separate file) Differential expression in 4-cell embryos resulting from naïve zygotes injected with sperm fragmented long RNA from control or MSUS males.

**References:**

1. Gapp, K. *et al.* Implication of sperm RNAs in transgenerational inheritance of the effects of early trauma in mice. *Nat. Neurosci.* **17,** 667–9 (2014).
